# Impact of Chromatin on HIV Replication

**DOI:** 10.3390/genes6040957

**Published:** 2015-09-30

**Authors:** Luis M. Agosto, Matthew Gagne, Andrew J. Henderson

**Affiliations:** 1 Department of Medicine, Boston University School of Medicine, Boston, MA 02118, USA; E-Mail: agosto@bu.edu; 2 Graduate Program in Microbiology, Boston University School of Medicine, Boston, MA 02118, USA; E-Mail: patsfan@bu.edu

**Keywords:** HIV, chromatin, integration, transcription, latency

## Abstract

Chromatin influences Human Immunodeficiency Virus (HIV) integration and replication. This review highlights critical host factors that influence chromatin structure and organization and that also impact HIV integration, transcriptional regulation and latency. Furthermore, recent attempts to target chromatin associated factors to reduce the HIV proviral load are discussed.

## 1. Introduction

Retroviruses such as the Human Immunodeficiency Virus (HIV) are defined by their ability to integrate their reverse transcribed genome into the host DNA prior to proviral transcription by the cellular machinery. The role of chromatin in early HIV infection and replication has been extensively studied, revealing that chromatin influences HIV integration as well as the establishment and maintenance of a transcriptionally repressed but inducible reservoir of cells infected with HIV. These latently infected cells present a barrier to curing HIV infection since they are a persistent source of virus following the interruption of antiretroviral treatments. Recent eradication strategies have focused on developing novel compounds and testing new indications for existing drugs that target chromatin remodeling factors. This review will examine the effects of chromatin on the HIV life cycle and discuss the potential of emerging treatments that target chromatin-modifying factors.

## 2. Chromatin and HIV Integration

Retroviral integration is not a random event and it has been estimated that 70% of HIV provirus is integrated into introns of actively transcribed host genes [1,2,3,4,5,6,7]. This bias in provirus location suggests that local chromatin organization influences the site selection of integration. Integration requires a combination of viral factors including capsid, reverse transcriptase and integrase, and host factors that facilitate the HIV pre-integration complex’s translocation into the nucleus and targeting the viral genome to the host DNA [8,9,10]. Several cellular factors have been identified as being a part of the pre-integration complex or to directly interact with HIV integrase [10,11]. These cellular factors may facilitate nuclear translocation of the preintegration complex through the nuclear pores, or influence targeting of the HIV proviral DNA by interacting with chromatin factors. However, few of these proteins have been validated as playing a role in establishing HIV provirus.

The best studied cellular factor that participates in HIV integration is LEDGF/p75 [12,13,14,15]. LEDGF/p75 is encoded by the PSIP1 gene and has been described as a transcriptional activator and a splicing factor, although, exactly how this relatively ubiquitous transcription factor functions in regulating gene expression is not clear [13,16,17,18,19,20,21]. LEDGF/p75 is part of a larger family of transcription factors, the hepatoma-derived growth factor-related proteins, which are defined by conserved N-terminal PWWP and A/T hook domains [22,23]. A/T hook domains mediate LEDGF/p75 non-specific binding [24] whereas the PWWP motif recognizes specific chromatin marks such as acetylated and methylated lysines in histone tails [25]. LEDGF also contains a C-terminal integrase binding domain (IBD) [26,27,28], which directly interacts with a binding pocket formed by two HIV integrase catalytic core domains (CCD) [28,29]. LEDGF/p75 involvement in HIV integration was initially suspected based on its interaction with integrase [29,30,31] and knockdown experiments validated the role of LEDGF/p75 in HIV infection and integration [32,33,34,35,36]. These studies demonstrated that although depletion of LEDGF/p75 modestly reduced HIV infection, there was an altered integration pattern with significantly reduced HIV integration into actively transcribed genes and a shift towards promoters and CpG islands being targeted by the provirus [9,14]. Structure and function studies are consistent with a model that LEDGF/p75, through its interactions with chromatin, tethers the HIV integrase dimer to the targeted DNA [24,25,29,37,38,39,40]. LEDGF/p75 may also promote integration by protecting HIV integrase from degradation and by stimulating its catalytic activity [24,30,41,42,43]. Recently, factors that interact with LEDGF/p75, including TOX4, NOVA1 [44], JPO2 [41,42,45] and PogZ, have been implicated in HIV integration [46]. In addition to facilitating HIV integration, LEDGF/p75 may silence HIV transcription and contribute to the establishment and maintenance of HIV latency by forming a complex with Spt6 and Iws1 that promotes the recruitment of repressive chromatin to the HIV promoter [47]. Other chromatin interacting proteins may compliment or even be functionally redundant to LEDGF/p75. For example, hepatoma-derived growth factor-related protein 2 (HRP2), which shares homology with LEDGF/p75, binds HIV integrase and rescues LEDGF/p75 activity in knockout cells [31,48,49]. Furthermore, LEDGF/p75 is not a general requirement for retroviral integration. Gammaretroviruses, such as MLV, preferentially integrate into strong transcriptional cis-elements including promoters, enhancers, and DNase hypersensitive sites and this bias is in part mediated by Bromodomain and Extra Terminal (BET) family transcription factors [8,9,50,51]. This indicates that selection of retroviral integration is specific to different retroviruses and influenced by the cellular cofactors that are usurped by the retroviruses.

Ini-1/hSNF5, a SWI/SNF ATP dependent chromatin remodeling factor, has also been suggested to influence HIV replication. The actual function of Ini-1/SNF5 in the context of HIV replication is still unclear, which may reflect the fact that Ini-1/SNF5 has multiple activities at different steps in the HIV life cycle [52,53,54,55]. Ini-1/SNF5 was originally identified as an HIV integrase-interacting partner using a yeast two-hybrid system [56] and was shown to be recruited into HIV virions and pre-integration complexes through a Gag mediated interaction [54,57]. *In vitro* assays show that Ini-1/hSNF5 facilitates integration by ATP-dependent displacement of nucleosomes to increase DNA accessibility [58]. In addition, disrupting Ini-1/SNF5-integrase interactions has been reported to alter integration [59,60]. Ini-1/SNF5 has also been suggested to have antiviral activities, inhibiting early steps of HIV replication through its interaction with HIV integrase as well as components of the Sin3a-HDAC1 repressor complex [52,54,59]. The functional relevance of Ini-1/SNF5-HIV integrase interactions warrants further investigation.

Recently, the TRIM family protein TRIM28/KAP1 has been shown to physically interact with and inhibit HIV-1 integrase [61]. TRIM28/KAP1 is a transcriptional co-repressor that interacts with KRAB zinc finger DNA proteins through its bromodomain, binding acetylated lysines to mediate gene silencing by recruiting repressor complexes to target promoters [62]. HIV integrase activity is regulated by p300 which acetylates key lysines K264, K266 and K273 within HIV integrase and TRIM28/SNF5 binds these acetylated lysines. TRIM28/KAP1 inhibits integrase activity by recruiting HDAC1 which deacetylates the lysines [61]. Therefore, Trim28/KAP1 coordinates HIV-1 integrase function with chromatin remodeling during proviral integration [61].

Using fluorescence *in situ* hybridization, immunohistochemistry and microscopy, it has been shown that the nucleus is highly organized and that genes are not randomly distributed, but are partitioned into regions that cluster expressed genes from repressed genes [63,64]. For example, transcriptionally active euchromatin is detected near nucleopore complexes, whereas transcriptionally repressed heterochromatin is enriched in areas between nucleopore complexes [65,66]. This chromatin architecture underneath the nuclear envelope is maintained by nuclear pore complex proteins including Nup153 and Tpr [67]. If expressed genes are juxtaposed to nucleopore complexes then, upon entry into the nucleus, HIV will most likely encounter actively transcribed genes with open chromatin. Furthermore, disrupting nuclear pore complexes would be predicted to compromise nuclear entry and/or integration of the HIV pre-integration complex. The pre-integration complex, HIV capsid and HIV integrase have been shown to interact with several nuclear pore proteins including transportin 3 and nucleoporins NUP153 and NUP62 [68,69]. In addition, a siRNA screen showed that targeting proteins associated with nuclear pore complexes, such as RANBP2, altered the selection of provirus integration sites [70,71]. Other nuclear pore proteins that mediate HIV infection include Nup153 and Nup98, which facilitate nuclear import of the preintegration complex and Tpr, which influences integration site selection by maintaining chromatin architecture, anchoring Nup153 and functionally associating with LEDGF/p75 [67,72,73,74,75,76].

Two recent reports support the possibility that HIV provirus interacts with batteries of actively expressed genes upon translocation into the nucleus. Firstly, it has been observed that HIV genomes co-localize with PML [77]. It is also interesting to note that PML functionally interacts with Ini-1/SNF5 [78]. Secondly, examination of integration sites by three-dimensional immune DNA fluorescence *in situ* hybridization suggested that integrated virus was strongly associated with chromatin regions at the outer region of the nucleus whereas integration was disfavored in lamin-associated domains located more centrally in the nucleus [79]. This bias in integration sites was dependent on Nup153 and LEDGF/p75. Most importantly, these studies demonstrate the role DNA topography plays in biasing HIV integration site selection [79].

## 3. Chromatin and HIV Transcription

In addition to influencing sites of integration chromatin architecture directly affects the efficiency of HIV gene expression and silencing. HIV transcription is initiated at the transcriptional start site within the R region of the 5' long terminal repeat (LTR) [80,81,82]. Efficient transcription of proviral DNA is tightly linked to the activation status of the cell and the availability of cellular transcription factors [83,84,85,86]. These factors recognize specific sequences within the HIV LTR and subsequently recruit the RNA polymerase II (RNAP II) transcriptional machinery. Initial transcription of the HIV provirus leads to the production of the virally encoded transcription trans-activator Tat. Tat significantly enhances the efficiency of transcription by binding a RNA stem loop at the beginning of the newly transcribed HIV RNA at the trans-activation response element or TAR. Tat recruits transcription elongation factors to the HIV promoter to facilitate provirus transcription. One critical factor recruited by Tat includes the P-TEFb complex, which phosphorylates the C-terminal domain of RNAP II as well as factors that act as negative elongation factors such as DSIF and NELF, enabling efficient processivity and elongation of the RNA transcript [87,88,89,90]. However, not all of infected cells support productive viral replication. In T cells, a subset of infected cells carries latent proviruses which are not efficiently expressed [80,91,92]. This pool of latently infected resting T cells represent an important barrier to curing HIV infection as these cells can be re-activated to produce virus at any point after the interruption of HAART and lead to the rebound of the viral load in infected individuals [93,94,95]. The transcriptional repression observed in latently infected cells may result from multiple mechanisms including low levels of positive transcription factors, the presence of repressive transcription factors, and epigenetic changes associated with chromatin that limit HIV transcription.

**Figure 1:**
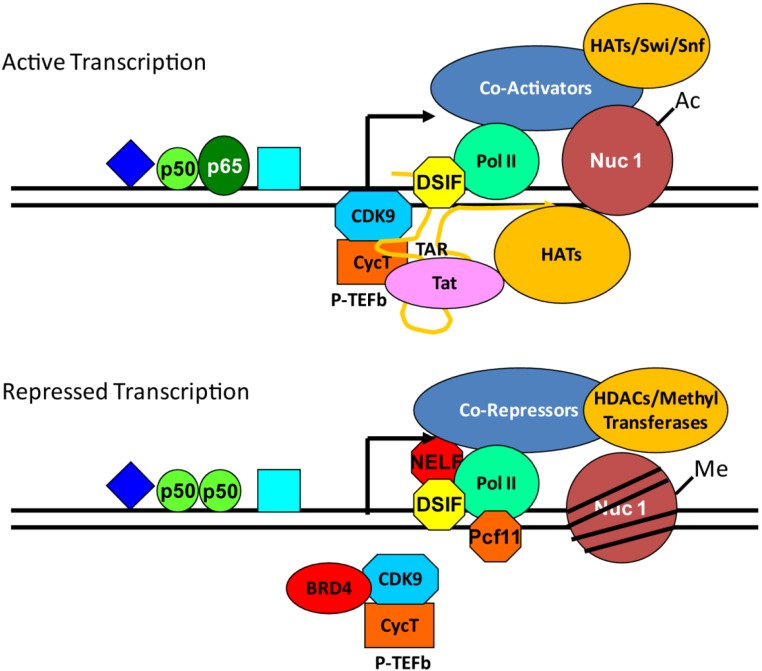
General regulation of HIV transcription and chromatin remodeling. Cellular transcription factors (e.g., p50/p65 NF-κB heterodimers) recognize motifs within the HIV provirus promoter region and recruit cellular co-activators, chromatin remodeling factors and transcription elongation factors. Negative transcription factors (e.g., p50/p50 NF-κB homodimers) recruit cellular co-repressors, chromatin remodeling factors and factors that hinder transcription elongation.

The proviral chromatin architecture includes two nucleosomes, designated as nuc-0 and nuc-1, located immediately upstream of the modulatory region within U3 and downstream of the transcription initiation site, respectively. The location of these nucleosomes were originally mapped in DNAse hyper-sensitivity studies [96]. This organization showed that the upstream cis-elements of the HIV LTR which include Sp-1, NF-κB and Ap-1 sites, are accessible and nucleosome free, whereas Nuc-1, in particular, is in a position to impede RNAP II elongation. The recruitment of histone-modifying complexes targets Histone-3 (H3) and Histone-4 (H4) of Nuc-1 to regulate HIV transcription [97,98,99]. A well characterized modification is acetylation of histones. Acetylation of histones by acetyltransferases (HATs) generally leads to opening of chromatin allowing accessibility of the DNA to the transcription machinery or enhanced RNAP II processivity. Cellular transcription factors including NF-AT, Sp1, NF-κB, AP-1, as well as HIV Tat recruit histone acetyltransferases such as CBP/p300, P/CAF and GCN5 to the HIV LTR (Figure 1). Acetylation of H3 and H4 of nuc-1 has been implicated in the activation of HIV transcription [100,101,102,103]. ChIP-analysis on the acetylation pattern of the nucleosomes following phorbol-ester stimulation of latently infect T cells showed increase acetylation of H3 at lysines 9 and 14 and of Histone-4 at lysines 8 and 16 [101]. Conversely, repressive transcription factors such as NF-κB p50 homodimers, YY-1, LSF, NELF and CBF-1 recruit histone de-acetylases (HDAC), leading to a closed chromatin conformation, limited access to the transcription machinery and decreased RNAP II activity [104] (Figure 1). These modifications are closely associated to the activation state of the host cell and are important for the silencing of HIV transcription and the establishment of latency. HDAC-1 [104,105,106,107,108,109], HDAC-2 [108,110,111] and HDAC-3 [88,110,112] are the main factors that mediate the de-acetylation of the nuc-1 of HIV as evidenced by ChIP analysis of repressive protein complexes, siRNA knock-downs and pharmacological inhibition. In addition to promoting opening and closing of the chromatin structure, the acetylation status of histones provides a platform for the association of additional regulatory proteins. One such protein is BRD4, which recognizes acetylated histones and transcription factors through its bromodomains. BRD4 recruits and activates P-TEFb through its P-TEFb-interacting domain (PID) [113,114]. The interaction of these proteins mediates basal transcription of the provirus in the absence of Tat. Interestingly, BRD4 competes with Tat for P-TEFb, thus acting as an antagonist for efficient proviral transcription elongation. BRD4 further antagonizes Tat transactivation by phosphorylating the CDK9 subunit of P-TEFb, thus inhibiting kinase activity within the HIV transcription initiation complex [115]. Based on these observations BRD4 has been suggested to be a critical factor for limiting HIV transcription and has been suggested to be a target for reactivating latent HIV (see below).

Histones are also modified by methylation and this modification, depending on the context and residue, either contributes to repression or to activation of transcription. Sp1 in complex with CTIP2 recruits SUV39H1 and mediates tri-methylation of H3 at lysine 9 [108]. The methyltransferase G9a also mediates repression of HIV transcription through dimethylation of H3 [116]. It remains unclear which factors mediate activation-related methylation, but it is possible that trimethylation of H3 at lysine 4 and dimethylation at lysine 36 mediated by SET1 and Smyd2 can promote proviral transcription [103,117,118,119]. Methyltransferases have also been shown to modify proviral DNA and HIV Tat creating additional layers of transcriptional regulation [120,121,122,123].

Another important modification to the chromatin structure is mediated by the SWI/SNF complexes. This is a large complex composed of 12 individual proteins [124]. The main function of SWI/SNF is to facilitate the mobilization of nucleosomes along the DNA in an ATP-dependent manner [124], thus facilitating access of DNA to the transcription machinery. In the context of HIV replication, SWI/SNF proteins influence integration (see above) and activate HIV transcription. Upon activation of the cell, Tat becomes acetylated. This modification of Tat allows it to bind to the catalytic subunit of the SWI/SNF complex known as Brg-1, thus recruiting the complex to the HIV promoter. The recruitment of SWI/SNF to the HIV promoter may also be facilitated by the interaction of Brg-1 with acetylated nuc-1 upon phorbol ester stimulation [125].

Chromatin re-assembly factors have also been proposed as limiting HIV transcription. Knockdown experiments of Spt6, Spt16 and Chd1, members of the facilitates chromatin transcription (FACT) complex, induced HIV transcription in latently infected cell lines [126]. These results suggest that the FACT complex promotes silencing of HIV transcription by assembling nucleosomes along the proviral DNA. Further studies are required to confirm the direct recruitment of these factors to the provirus and how their activity is regulated in the context of HIV latency.

## 4. Targeting Chromatin as a Therapeutic Strategy for HIV

Given the importance of DNA architecture for the efficient integration and transcription of HIV, chromatin modifying factors have been appealing targets for anti-retroviral therapies (Table 1). To block early steps in the HIV life cycle, drugs modulating the interaction between LEDGF/p75 and HIV integrase (IN), called LEDGINs, are under development. Christ *et al.* identified 2-(quinolin-3-yl) acetic acid compounds that inhibited binding between HIV integrase and LEDGF/p75 [127]. Furthermore, these LEDGINs stabilize IN multimers, allosterically inhibit integrase activity and prevent proper virion assembly [127,128,129,130]. Although the initial LEDGINs displayed IC_50_ values in the micromolar range, tert-butoxy-(4-phenyl-quinolin-3-yl) acetic acid derivatives are potent in the nanomolar range, increasing the likelihood that they will be brought to clinical trials [128].

Chromatin-targeting strategies for treating HIV also include compounds designed to work at the level of transcription such as “shock and kill” strategies to reactivate latent proviruses in conjunction with antiretroviral therapy [131]. With virus replication blocked by routine antiretroviral therapy, it is expected that the reactivated latent population will be cleared by the host immune system or by the cytopathic effects of viral replication. Many of these latency-reversing agents (LRAs) target regulators of chromatin.

**Table 1:** Potential HIV therapeutics that target chromatin.

Drug Class	Compounds	References
**Inhibitors of Integration**		
LEDGINs	2-(quinolin-3-yl) acetic acids, tert-butoxy-(4-phenyl-quinolin-3-yl) acetic acids	[127,128,129,130]
**Transcriptional Activators**		
Histone Deacetylase Inhibitors (HDACi)	Valproic acid, SAHA, (vorinostat), givinostat, panobinostat, entinostat, romidepsin	[132,133,134,135,136,137,138,139,140,141,142,143,144,145,146,147,148,149]
BET Bromodo main Inhibitors	JQ1	[150,151,152,153,154]
Histone Methyltransferase Inhibitors (HMTi)	Chaeotocin, BIX-01294, DZNep	[155,156,157]
DNA Methyltransferase Inhibitors (DMTi)	5-aza-2'-deoxycytidine	[121,122,157]

The most widely studied LRAs are HDAC inhibitors, which maintain an open chromatin structure by histone acetylation. The epilepsy drug, valproic acid (VPA), is a Class I/II HDAC inhibitor [132]. Phase I and II clinical trials for using VPA as a latency-reversing agent have so far shown mixed results regarding changes in the amount of integrated virus as measured by PCR. In the initial proof-of-concept study, three out of four patients obtained a mean reduction of 75% infected units per billion (IUPB) as determined by a viral outgrowth assay (VOA) [133]. A follow-up trial was only able to replicate a statistically significant decrease in IUPB for four out of 11 subjects [134]. In addition, subsequent studies indicated that valproic acid did not alter the amount of integrated latent HIV [135,136,137] and may increase the cognitive impairments associated with AIDS [138].

SAHA or vorinostat, a treatment for T cell lymphomas, is another promising HDAC inhibitor. It induces HIV expression in cellular models of HIV latency, such as J-LAT, J∆K, ACH-2, and U1 cells, as well as *ex vivo* experiments with patient peripheral blood mononuclear cells [139,140,141]. In a small clinical trial, SAHA treatment substantially increased cell-associated viral RNA levels in patients on ART, several of whom had no detectable HIV RNA before study enrollment [142]. However, a second clinical trial demonstrated that, although oral doses of vorinostat led to a significant increase in cell-associated unspliced HIV RNA for the majority of subjects, there was no change in plasma HIV RNA or the amount of integrated HIV DNA [143].

A large number of other HDAC inhibitors are under investigation, including givinostat, panobinostat, entinostat, and romidepsin [144,145,146,147,148]. Rasmussen *et al.* [145,149] contrasted the effects of clinically relevant concentrations of panobinostat, givinostat, vorinostat, and valproic acid on U1 and ACH2 latent cell line models as well as latently-infected resting primary cells. Panobinostat, a hydroxamic-acid pan-HDAC inhibitor, significantly increased HIV transcription as compared to the other inhibitors for all cell types [145]. A clinical trial with panobinostat demonstrated its capacity to upregulate cell-associated unspliced HIV RNA and temporarily perturb the amount of viral DNA in patients on ART [149].

Besides HDAC inhibitors, several groups have shown that the BET bromodomain inhibitor JQ1 can reactivate HIV in U1, ACH2, J∆K, and J-Lat cell models [150,151,152,153,154]. In addition, JQ1 induced HIV expression *ex vivo* in T cells from one out of three individuals on ART [150]. Evidence suggests that JQ1 may inhibit the BET protein BRD4, which represses transcription by sequestering PTEF-b from Tat [113,151]. However, recent studies suggest that JQ1 may function by inhibiting BRD2. Although the mechanism by which BRD2 contributes to HIV latency has not been elucidated, Boehm *et al.* postulated that BRD2 may target HDACs to the proviral LTR [154]. JQ1 has not yet been brought to clinical trials for the treatment of HIV.

Histone methyltransferases (HMTs) present another potential target for LRAs. Chaeotocin, a fungal toxin and SUV39H1 HMT inhibitor (HMTi), reactivates HIV in latently infected cell models [155,156]. Furthermore, chaeotocin and BIX-01294, a G9a HMTi, can stimulate HIV transcription *ex vivo* in PBMCs from ART-suppressed HIV^+^ patients. In contrast, other evidence indicates that DZNep, which inhibits several HMTs including EZH2, is a more potent inducer of HIV transcription than either chaeotocin or BIX-01294 in latently infected Jurkats [157]. These HMTis, however, are too toxic to be used as single agents in clinical trials. DNA methyltransferase inhibitors, while also appealing, have not yet demonstrated the same potential to induce latent HIV expression as HMTi’s. 5-aza-2'-deoxycytidine, which inhibits the cytosine methylation associated with CpG islands, weakly reactivates HIV but can synergize with other LRAs such as the protein kinase C modulator prostratin [121,122,157].

In spite of promising results obtained with cellular models of latency and *ex vivo* activation of CD4^+^ T cells from HIV patients, LRAs have had little success in clinical settings. Other than early trials of valproic acid in combination with ART [133], none have successfully reduced the viral reservoir *in vivo*. However, the potential for global T cell activation, specifically for HDAC inhibitors, may have had significant negative repercussions within these trials and would have limited the dosages, perhaps below the therapeutic threshold. This has led to the search for non-T cell stimulating agents that reactivate latent HIV, like protein kinase C activators. These compounds, which use mechanisms of action unrelated to chromatin dynamics, may have increased efficacy after other LRAs have reorganized the DNA architecture. Indeed, several studies have identified that combinatorial approaches maximally induce HIV expression in latently infected primary cells and cell models [43,140,152,153,157,158,159]. For example, it was demonstrated that several minimally effective small molecules synergize with known activators such as prostratin to induce HIV expression in J-Lat cells [160].

Multifaceted strategies to induce HIV expression will be essential to significantly perturb the HIV reservoir. New evidence indicates that we may be substantially underestimating the size of the viral reservoir. With VOA-based measurements, about one in 10^6^ resting CD4^+^ T cells are estimated to be inducible [161,162]. In contrast, genomic PCR indicates that nearly 200 in 10^6^ resting CD4^+^ T cells contain integrated provirus [163] and this proviral load may be dynamic and vary between patients. It has been assumed that the majority of these integrated proviruses are replication-incompetent. However, the Siliciano lab sequenced 213 proviruses from eight patients on ART that were incapable of being activated by the standard LRA phytohemagglutinin and showed that approximately 10% of proviruses deemed non-inducible by VOA were actually capable of infection and replication [164]. Therefore, with this uncertainty surrounding the size and location of the latent reservoir, the potential of LRAs may need to be further evaluated.

Future design of HIV/AIDS therapeutics must incorporate drugs designed to work at the level of chromatin. Host and proviral genomic architecture have profound repercussions for the fate of infected cells. Specific manipulation of chromatin would allow us to modify the balance between the maintenance of latently infected cells and cells containing transcriptionally active proviruses that can then be targeted and finally cleared.

## 5. Conclusions

Chromatin influences HIV integration and transcription which has implications for efficient HIV replication and latency. Therapeutically targeting chromatin and associated factors provides promising strategies for preventing HIV infection and reducing the size of the latent reservoir.
